# Inaccuracies in calculating predicted body weight and its impact on safe ventilator settings

**DOI:** 10.1186/2197-425X-3-S1-A685

**Published:** 2015-10-01

**Authors:** I OBrien, A Middleditch, C Bigham

**Affiliations:** Plymouth Hospital NHS Trust, ICU, Plymouth, United Kingdom

## Introduction

Lung protective ventilation has been widely accepted as a standard of care in patients with ARDS.([1]) The benefit to all ventilated patients in the ICU is less clear.([2]) An informal survey of the southwest region of the UK confirmed that on admission of a ventilated patient, most units measure patient height using a 1 m tape, derive predicted body weight (PBW) and convert this to tidal volume (TV) using 6 to 8 mls/kg. Many units audit their adherence to low tidal volume ventilation. However, there are no reports of the accuracy of estimating predicted body weight in supine unwell patients on which these lung protective strategies are based.

## Objectives

The aim of this project was to assess the accuracy and precision of patient measurement and PBW estimation in our unit and to evaluate alternative techniques.

## METHODS AND RESULTS

**Figure 1:**
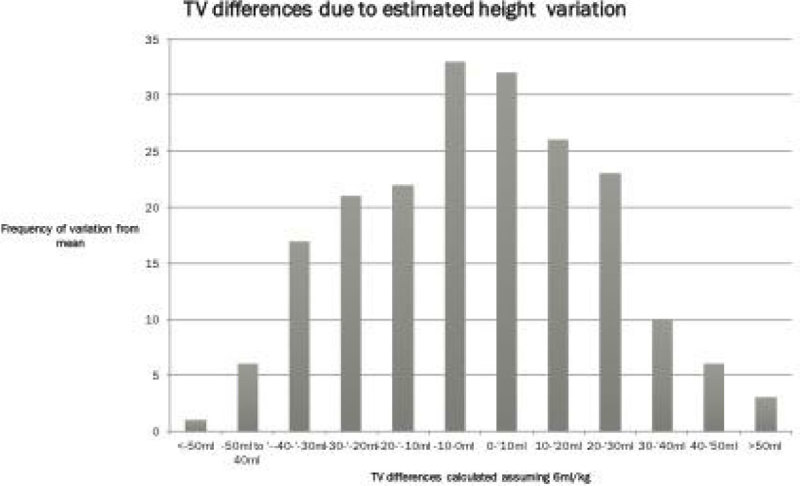
Figure 1

As a snapshot of current practice, a random sample of 20 nurses were asked to measure 20 patients using their normal technique and our standard 1 m tapes, as is our normal practice. They were blinded to each other's measurements. There was a surprisingly high degree of variability in the measurements leading to differences in derived TV's. The largest TV difference from the mean TV calculated for a single patient was 63 mls (17% of mean TV for this patient) Figure 1 demonstrates the spread in TV values from the mean calculated for each patient.

We examined alternative techniques to derive PBW([3]):olecranon to ulnar styloid distance, knee to sole height and sternal notch to tip of middle finger. 21 members of nursing staff measured the predicted body weight of 8 subjects using the 4 different techniques. Individuals were blinded to other's measurements and standing height was used as a “gold standard” for comparison.

**Figure 2:**
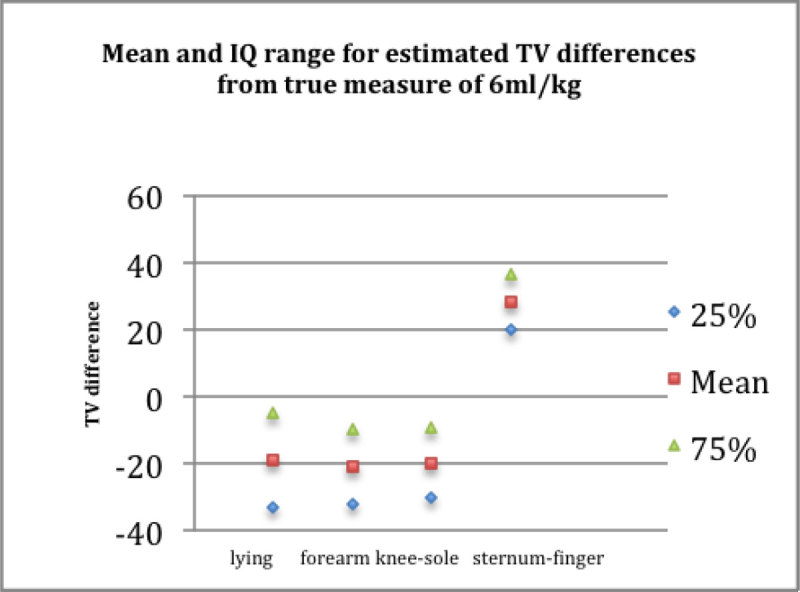
Figure 2

Figure 2 shows the TV difference and range of values generated by each modality as compared to the gold standard.

The data demonstrates that no technique is accurate to standing height measurement and that most underestimate true height. Sternum to middle finger is most precise but least accurate and the others broadly compare.

## Conclusions

Much work has gone into elucidating the optimal mls/kg for ventilation of critically unwell patients with great emphasis on getting the numerator right. Error in measurements used to estimate PBW have not received the same scrutiny. When used to generate TV's, these will be amplified by multiplication possibly leading to clinically significant higher TV settings. As a centre, we have invested in 2 m tapes and are considering laser measurers to improve our ventilation strategies.
